# Reduced Graphene Oxide UWB Array Sensor: High Performance for Brain Tumor Imaging and Detection

**DOI:** 10.3390/nano13010027

**Published:** 2022-12-21

**Authors:** Mohd Aminudin Jamlos, Mohd Faizal Jamlos, Wan Azani Mustafa, Nur Amirah Othman, Mohamad Nur Khairul Hafizi Rohani, Syahrul Affandi Saidi, Mohd Sharizan Md Sarip, Mohd Al Hafiz Mohd Nawi

**Affiliations:** 1 Faculty of Electronic Engineering Technology, Universiti Malaysia Perlis, Arau 02600, Malaysia; 2 Centre of Excellent for Advanced Communication Engineering (ACE), Universiti Malaysia Perlis, Arau 02600, Malaysia; 3 Faculty of Electrical and Electronics Engineering Technology, Universiti Malaysia Pahang, Pekan 26600, Malaysia; 4 Centre for Automotive Engineering Centre, Universiti Malaysia Pahang, Pekan 26600, Malaysia; 5 Faculty of Electrical Engineering Technology, Universiti Malaysia Perlis, Arau 02600, Malaysia; 6 Faculty of Chemical Engineering Technology, Universiti Malaysia Perlis, Arau 02600, Malaysia; 7 Faculty of Mechanical Engineering Technology, Universiti Malaysia Perlis, Arau 02600, Malaysia

**Keywords:** reduced graphene oxide (RGB), UWB array, microwave imaging

## Abstract

A low cost, with high performance, reduced graphene oxide (RGO) Ultra-wide Band (UWB) array sensor is presented to be applied with a technique of confocal radar-based microwave imaging to recognize a tumor in a human brain. RGO is used to form its patches on a Taconic substrate. The sensor functioned in a range of 1.2 to 10.8 GHz under UWB frequency. The sensor demonstrates high gain of 5.2 to 14.5 dB, with the small size of 90 mm × 45 mm^2^, which can be easily integrated into microwave imaging systems and allow the best functionality. Moreover, the novel UWB RGO array sensor is established as a detector with a phantom of the human head. The layers’ structure represents liquid-imitating tissues that consist of skin, fat, skull, and brain. The sensor will scan nine different points to cover the whole one-sided head phantom to obtain equally distributed reflected signals under two different situations, namely the existence and absence of the tumor. In order to accurately detect the tumor by producing sharper and clearer microwave image, the Matrix Laboratory software is used to improve the microwave imaging algorithm (delay and sum) including summing the imaging algorithm and recording the scattering parameters. The existence of a tumor will produce images with an error that is lower than 2 cm.

## 1. Introduction

By 2030, it is estimated that almost 13 million individuals globally will be affected by cancer, which can be considered as one of the most complex illnesses that ever existed [1]. Early cancer detection might increase the survival ratio among the cancer patients due to the fact that less sever and more varied treatment options could be offered. Presently, Computed Tomography (CT) Scan, Magnetic Resonance Imaging (MRI), X-ray, microwave imaging, and the ultrasound machine have been used as current imaging equipment applied specifically to identify various types of cancers [2]. Among them, the microwave imaging technique for cancer detection offers numerous significant advantages as well as being firm, inexpensive, safe, and non-invasive [3,4]. The formation of tissue with an irregular cell development is well-defined as a brain tumor, which is considered to be one of the most complicated cancers ever to exist [5]. To visualize the internal human structure, microwave imaging is used in order to reveal electromagnetic fields that work in microwave frequencies, with a range of 300 MHz and 30 GHz [6]. There are three techniques used for microwave imaging, namely hybrid, active, and passive.

Application of microwave energy for brain tumor imaging for detection purpose is currently gaining interest by the research community. Microwave imaging systems have the high potential of being simple, safe, portable, and cost-effective. Nowadays, a variety of imaging modalities are used to diagnose brain tumor, such as conventional X-ray, CT- Scan and MRI. However, the current method is associated with ionizing radiation, invasive, with long scan duration, and very expensive [7]. Thus, new imaging modalities such as microwave imaging systems equipped with simple, fast, portable, safe, and cheap detectors need to be deployed for initial brain tumor detection.

The transmission of microwave signals into tissues is utilized by a sensor for active microwave imaging and the returned signals are collected to produce microwave images. Tomography and radar imaging are the active microwave imaging for detection purposes such as breast tumor detection that only can be detected by UWB radar imaging technique [8]. Moreover, tumors and strokes can also be detected by using the same procedures [9]. Simple methods such as radar-based techniques are employed as they only require recognition of the intense scattering point produced due to the presence of high recurrent signal reflections [10]. Thereby, the tumor may be identified via the delay and sum confocal approach for image processing algorithms [11]. Impressively, a confocal imaging approach that reconstructs microwave images from backscattered signals by delay and sum (DAS) algorithms as beam former has been utilized in this paper and significantly contributes to the reduction of clutter to create more intensity of microwave imaging [12].

Notably, g-C_3_N_4_ has developed into a novel family of next-generation visible-light-driven polymeric semiconductors that are non-toxic, metal-free, plentiful in the earth, and showing useful properties in organic waste degradation, hydrogen evolution from energy conversion, water, sensing, and imaging. Numerous reviews mostly focus on applications for g-C_3_N_4_ in catalysis and its production. The photo- and organic-catalyst, bioimaging, (chemical and bio-) sensing, electronics, and energy-related uses of g-C_3_N_4_ (batteries, supercapacitors, white-light-emitting diodes, and oxygen reduction process) have not yet received a systematic description. [13]

Lately, graphene is rapidly being used as the optional element for electronic devices including telecommunication devices [14]. Owing to various benefits of a patch sensor operating in the microwave frequency range, graphene has the high potential to substitute the common metal of copper. Graphene sensors perform better as compared with single-wall and multi-wall carbon nanotubes as well as copper patch sensors, as demonstrated by Moon et al. [15]. This is due to its properties, including being 0.33 nm in size, best conductivity (10^8^ s/m), lightness (0.77 mg/ m^2^) and strength (150,000,000 psi) due to the lattice structure of one-layer carbon atoms. Especially, the graphene sensor demonstrates higher conductivity (3.7 × 10^8^ s/m) than copper (3.7 × 10^7^ s/m) in the microwave frequencies. These vital properties of graphene serve as a promising alternative to patch sensor material for cancer detection purposes due to a higher microwave signal required for human head structure penetration, including skin and skull in order for the signal to reach the brain [16].

Although pure graphene is costlier, realization of low cost RGO originating from cheap graphite was made possible with the advancement of chemical technology where the synthesized low-cost RGO exhibits the same characteristics of the pure graphene [17]. The oxidation process of graphite in order to transform into graphene oxide (GO) has made the electrical conductivity properties of the graphene oxide (GO) disappear during the synthesis process. Hence, special reduction processes are required to restore its electrical conductivity [18]. The reduction procedure used in this work defines the final electrical conductivity and assures that it performs similarly to real graphene [19]. Almost all reduction procedures entail reactive chemicals employment, which, if not adequately controlled, can be hazardous for the user as well as the environment as a long-term effect. As a result, a unique technique is used to reduce graphene oxide using a non-hazardous chemical (Hydroiodic acid) and a two-step combination of chemical and microwave aided processes (RGO) [20].

The novel development of a low-cost with high-performance reduced graphene oxide (RGO) UWB array sensor for brain tumor detection is described in this paper. The utilization of the combined RGO is pointed toward improving sensor gain and bandwidth. It enables the infiltration into the complex human head through interaction at various frequencies, as such cancers are normally situated inside the brain [21]. Moreover, the Improved Delay and Sum (IDAS) imaging algorithm is introduced by applying *mslowess* filter as for the smoothing technique by reducing the clutter to create more intensity of microwave imaging.

## 2. Experimental

### Electrochemistry

**Figure 1:**
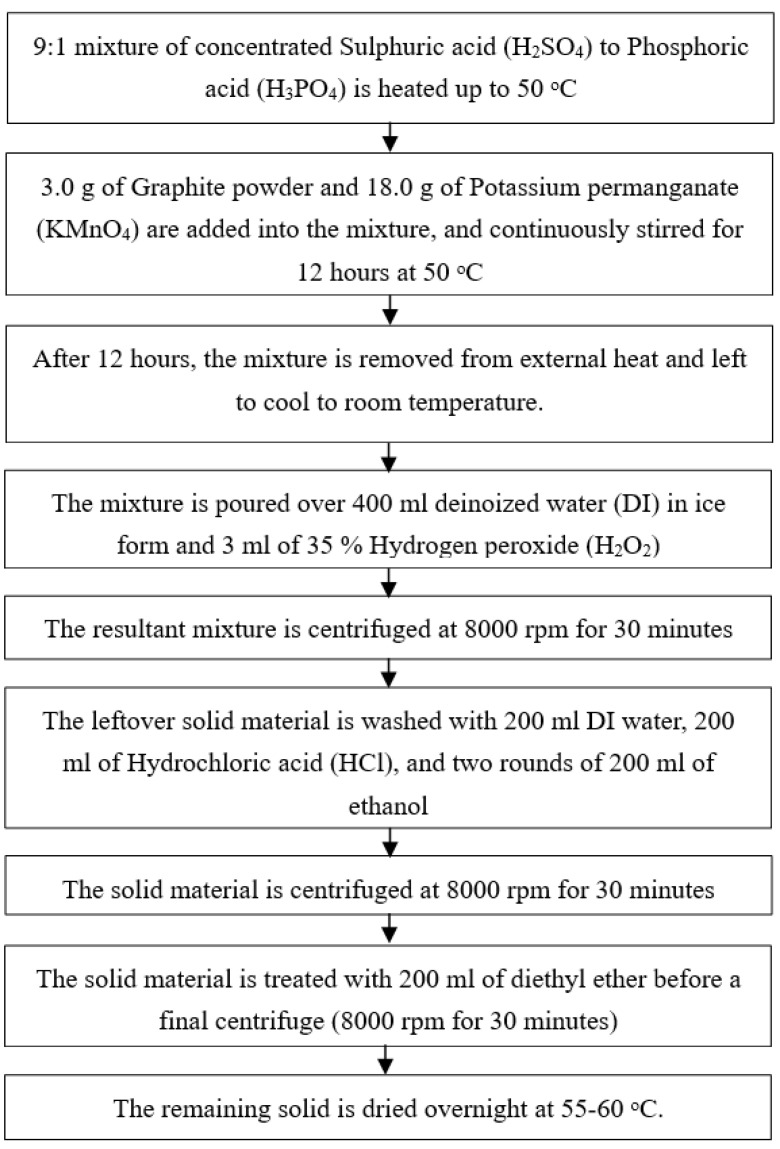
Graphene Oxide synthesizing process using Tour’s method.

**Figure 2:**
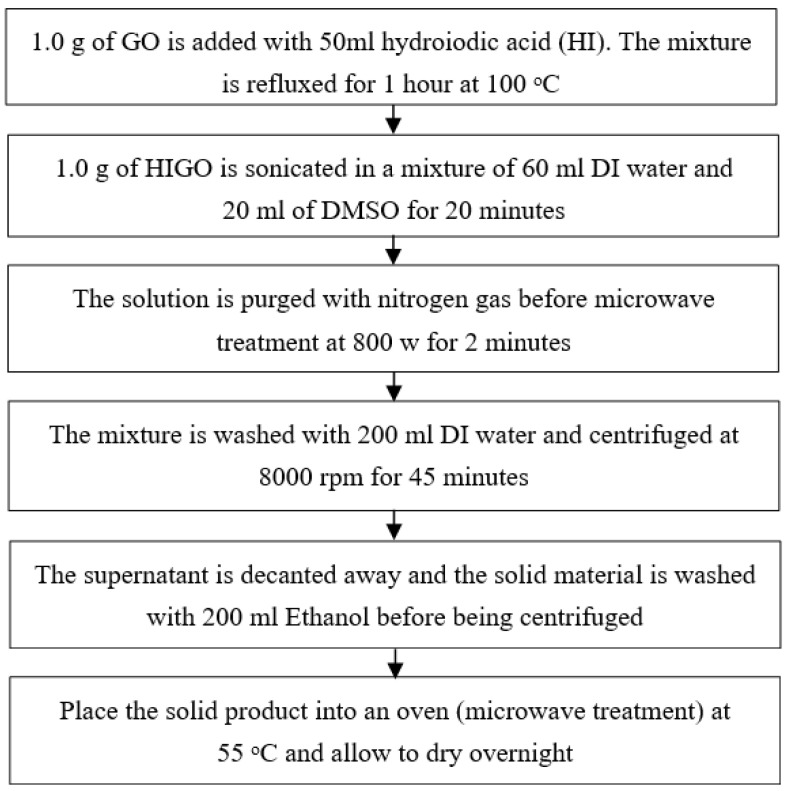
Reduced Oxide synthesizing using an alternative method of chemical and microwave combination for the reduction process.

Figure 1 and Figure 2 clarified exhaustively the preparing steps (flowchart) for incorporating the GO from Graphite to RGO utilizing microwave and chemical method separately. The chemicals are bought from Sigma-Aldrich.

## 3. Methodology and Experimental Set Up

### 3.1. Morphological Analysis from FESEM

**Figure 3:**
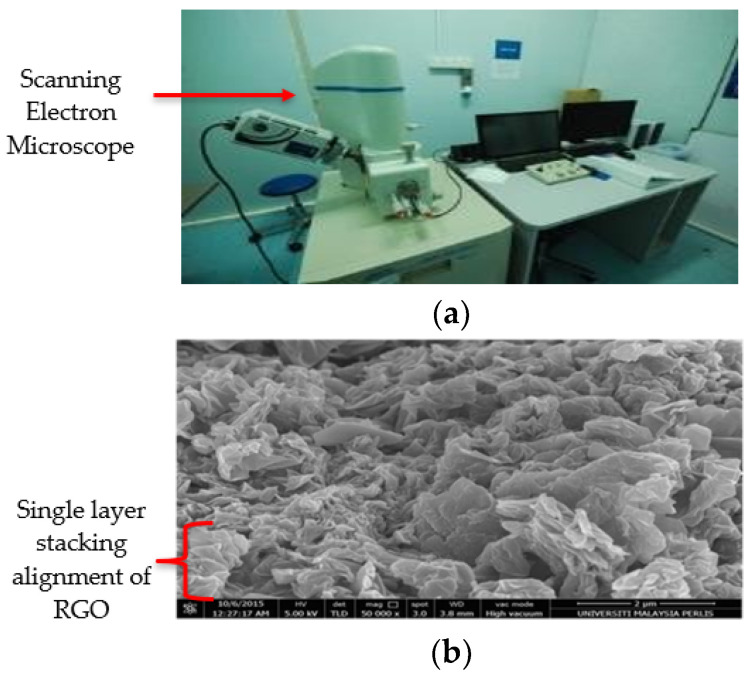
(**a**) Scanning Electron Microscope device (**b**) RGO FESEM image with 50 K of magnification.

Figure 3a,b shows Field Emission Scanning Electron Microscope device and RGO SEM image respectively. The figure clearly illustrates the formed of scattered groups RGO sheets with a clear sign stacking. The RGO sheet looks like a layer sheet morphology in excess of outsized area due to large surface to volume ratio. Similar morphological nature of the RGO with sheets closely stacked due to elimination of oxygen group to form a closely associated stack arrangement has been reported in literature [22].

### 3.2. Structure Study from XRD

**Figure 4:**
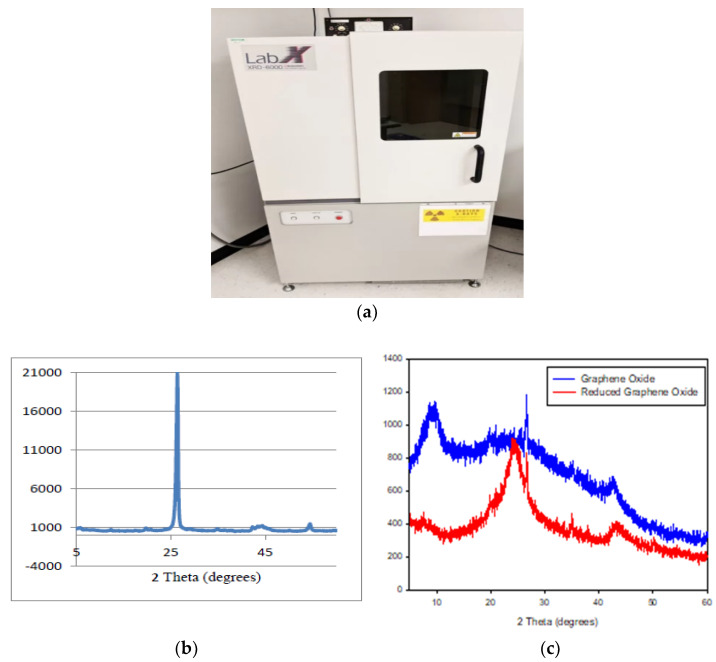
(**a**) X-ray Diffraction (XRD) device, XRD characterization; (**b**) pure graphite, (**c**) graphene oxide and reduced graphene oxide.

X-ray Diffraction (XRD) device and XRD patterns of pure graphite, GO an RGO are presented in Figure 4a, Figure 4b, and Figure 4c respectively. From Figure 4a, the most intensive characteristic peak observed at 26.60° corresponds to the (002) plane. It is clearly indicating the formation of pure graphite [23]. The reaction of oxidation in Figure 4b presents the broader diffraction peak at 12.70° which tends to wider graphite unique peak and moves closely to 12.70°. Moreover, it implies that the obtained diffraction shifts in GO due to the interface space higher than graphite [24]. In the case of RGO (Figure 4b), a new characteristic broad diffraction peak (broad hump) appears to be centered at 23.5°. An increase of broad humps due to reduction of oxygen functionalities point out to de-crease of intercalated layer sheets. The reductions of oxygen function groups induce an increase in interplanar distance. Similar work has been reported using reduction technique of functional group for oxygen removal by Wang et al. [25] where the stacking sheets are closely linked to each other due to the interplanar distance and d-spacing reduction. The formation of graphene single layer structures due to the removing of the multilayer component will increase the greater spreading peak. The decreasing of the size of sheets with a shapeless arrangement is formed from a broad hump at 2θ = 20–30° [26]. Thus, the two-stepped method of this study in producing RGO is proven to have sufficient reduction properties as compared to previous techniques reported in the literature [27]. The removal or reduction of oxygen functionalities due to this chemical with the microwave assisted combination process reduction method leads to an increase in conductivity of RGO.

### 3.3. Electrical Study of Four-Point Probe

**Figure 5:**
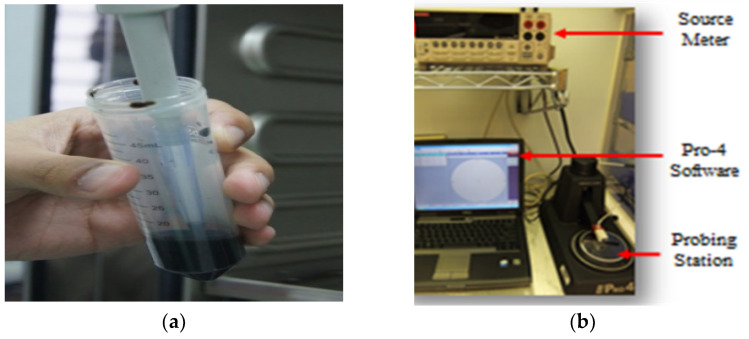
(**a**) Liquid reduced graphene oxide (**b**) Four-point probe device.

Figure 5a,b shows RGO in the liquid form and Four Point Probe device respectively. RGO recorded 3.38 × 10^8^ s/m (conductivity), 2.01 × 10^−3^ m (resistivity), 0.2 V (power), and 9.48 × 10^−1^ A (current) according to the experiments. The measured high conductivity and low resistivity values are similar as compared to the early findings on conductivity and resistivity of RGO by several researchers [25,26,27]. The observed electrical properties are sufficient to be utilized as conductive material to replace copper and other metals for various applications. The chemical with microwave assisted combination process-based reduction method is playing a prime role to enhance the electrical properties.

### 3.4. Sensor Design and Development

**Figure 6:**
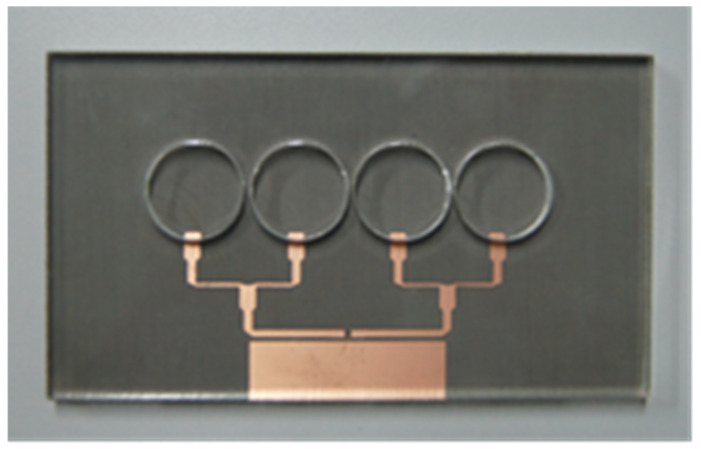
Perspex mould positioned above.

It is computed how much RGO is needed to make circular patches with a radius of 15 mm and thicknesses of 0.035. As shown in Figure 6, the patch positions are subsequently taken on top of the substrate by a bespoke mould. Once all four moulds are filled with liquid RGO, are heated for 5 h in hot oven with 50 °C. Excessive heating with longer time and higher temperature could compromise the structure of the RGO as the process needs to be performed with care as it may lead to cracked RGO layers.

**Figure 7:**
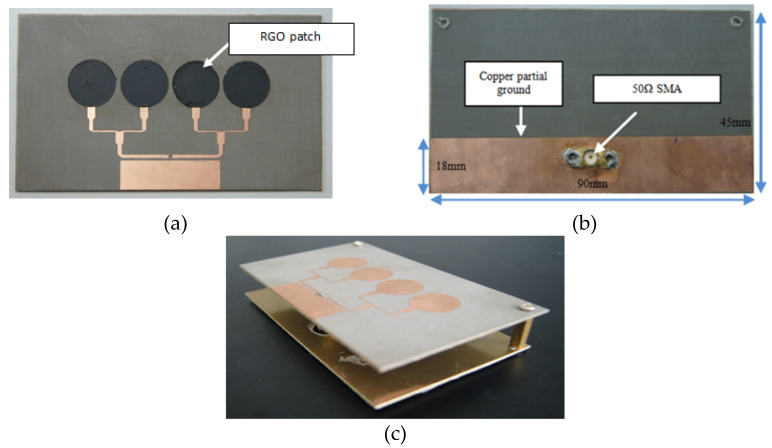
Fabricated sensor views; (**a**) front, (**b**) back, (**c**) side.

Figure 7 illustrates UWB RGO sensor with Taconic as the substrate which has characteristics of 1.58 mm in thickness, 0.0009 of tangent loss and 2.2 of dielectric constant. On the other hand, RGO construct the patches while parasitic element and transmission lines are constructed by common copper as shown in Figure 7a. The patches are equal, having diameter of 15 mm and the square parasitic element dimensions of 8.0 × 32.0 mm^2^, which is located adjacent to the transmission lines. It has Ultra-Wide Band characteristic through focusing the wave within desired direction by terminating the wave propagated in unwanted directions [28]. This contributes towards the novel design of the UWB RGO sensor. Meanwhile, Figure 7b shows the partial ground made of copper, which measures 18 × 90 mm^2^, as well as a Sub Miniature connection placement at the back of the substrate plane. This structure’s total dimensions are 90 mm × 45 mm^2^. In order to improve gain and directivity, a reflector made of copper has been placed 20 mm away behind the sensor to eliminate back lobes [29], which is proven by the high gains obtained and shown in Figure 7c. The reflector dimensions are similar to the sensor dimensions.

The physical dimensions of the sensor patch (*r*), a radius of 15 mm and thickness of 0.035, could be determined as follows [30]:

(1) $$r=\frac{F}{{\left\{1+\frac{2h}{\pi \varepsilon_{rF}}\left[ln\left(\frac{\pi F}{2h}\right)+1.7726\right]\right\}}^{1/2}}$$

(2) $$F=\frac{8.791\times 10^{9}}{f_{r}\sqrt{\varepsilon_{r}}}\;$$

where *r* = patch radius, *h* = substrate thickness, *fr* = resonant frequency, *Ɛr* = substrate dielectric constant, *Ɛeff* = effective dielectric constant.

Through side feeding, the proposed sensor was supplied by a 50 Ω SMA connection. The feed line is 2.9 mm in diameter to exactly match the sensor’s source feed of 50 Ω. The following is the formula for determining microstrip feed lines dimension to obtain impedance of 50 Ω [30]:

(3) $$\;W_{m}=\frac{120\pi }{\sqrt{\varepsilon_{r}}}$$

where *Wm* = microstrip feed line width, *h* = substrate thickness, *Ɛr* = substrate dielectric constant, *Z*_0_ = input impedance.

The parasitic element is electromagnetically attached to the radiating element to form passive resonator to increase bandwidth and efficiency by suppressing the surface current at the transmission line of the sensor which has a length of 32 mm and width of 8 mm for two closely situated radiating elements [31]. To establish the sensor’s radiation and ability to cut off the waves in an unwanted direction, the waves from the parasitic elements should not interfere. The quarter-wave converter technique for current matching purpose ensures the same amount of current is received by the entire RGO patches. Then, 70.71 Ω quarter-wave converters were utilized to perfectly match the impedance lines of 100 and 50 Ω. The quarter-wave converter’s formula is presented in the following equation [32]:

(4) $$Z_{1}=\sqrt{Z_{0}R_{in}}$$

where *Wm* = microstrip feed line width, *h* = substrate thickness, *Ɛr* = substrate dielectric constant, *Z*_0_ = input impedance, *Z*_1_ = transformer characteristic impedance, *R_in_* = edge resistance at resonance.

Equations (5) and (6) are used to calculate the width of the impedance feedlines (50 and 100) as well as quarterwave transformer (70.71) [32]:

(5) $$\mathrm{For}\;\frac{W}{h}<2,\;\frac{W}{h}=\frac{8e^{A}}{\left(e^{2A}-2\right)}$$

(6) $$\mathrm{For}\;\frac{W}{h}<2,\;\frac{\;W}{h}=\frac{2}{\pi }\left[B-1-\ln\left(2B-1\right)+\frac{\varepsilon_{r}-1}{2\varepsilon_{r}}\left\{\ln\left(B-1\right)+0.39-\left(\frac{0.61}{\varepsilon_{r}}\right)\right\}\right]$$

where *h* = substrate height while *A* and *B* could be evaluated as follows:

(7) $$A=\frac{Zo}{60}{\left[\frac{\varepsilon_{r}+1}{2}\right]}^{\frac{1}{2}}+\left[\frac{\varepsilon_{r}-1}{\left(\varepsilon r+1\right)\left(0.23+\left(\frac{0.11}{\varepsilon_{r}}\right)\right)}\right]$$

(8) $$B=\frac{377\pi }{2Z_{0}\sqrt{\varepsilon_{r}}}$$

### 3.5. Sensor Principle of Operation

**Figure 8:**
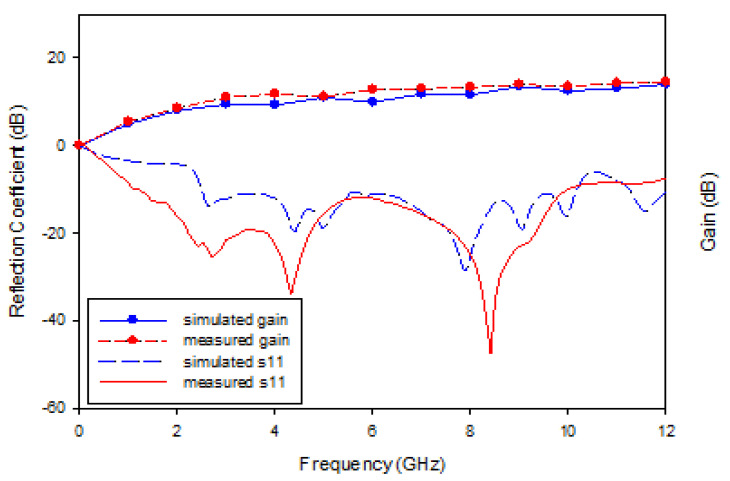
S11 result with gain for the sensor.

The UWB RGO sensor exhibits operated frequency of less than −10 dB ranging from 1.2 GHz–10.8 GHz for measured while 2.1–10.2 GHz for simulated with enormous energy generated ranging from 5.2–14.5 dB for measured gain and 5.0–14.0 dB for simulated gain, as demonstrated by Figure 8. The different results of measured and simulated reflection coefficient (s11) are due to deficiency of RF cable which connected between sensor and vector network analyzer (VNA) during the measurement. Yet, the operated frequencies are still within the desired frequencies for both simulated and measured. The simulations were performed in Microwave Studio by CST, a full wave electromagnetic solver, specifically using time solver. For the radiation safety part, the sensor radiated microwave energy is set to be less than 1.6 W/Kg for Specific Absorption Rate standardized by Federal Communications Commission (FCC). The value recorded by the sensor is 1.2 (W/Kg).

**Figure 9:**
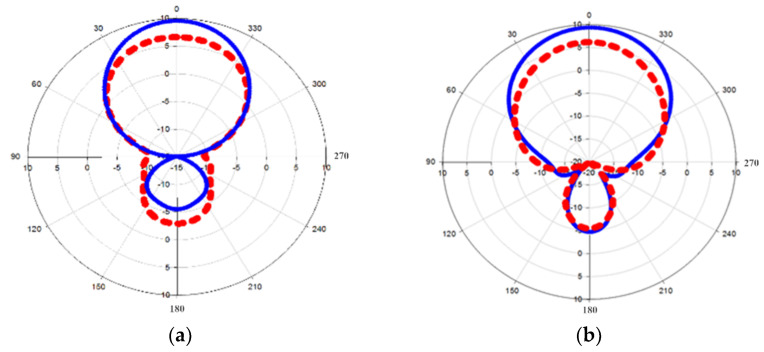
Azimuth plane of polar radiation pattern in: blue (measured) and red (simulated), (**a**) 2 GHz and (**b**) 3 GHz.

Figure 9 exhibits the sensor’s polar energy form in the azimuthal direction for microwave brain imaging frequency resonance at 2 GHz and 3 GHz. Basically, both simulated and measured radiation patterns do radiate in a wide frequency band and could be considered as a unidirectional sensor due to the radiated wave continuously radiating toward the main lobe as compared to the side and rear lobes [33,34]. Moreover, the results of simulation and measurement indicate a satisfactory agreement. All the characteristics such as sensor wave shape, wide operating frequency, and high gain have made the sensor an ideal sensor for brain tumor detection.

### 3.6. Sensor Integration and Experimental Test

Interaction of electromagnetic waves in the frequency band of 0.3 to 30 GHz with bio-logical tissues could lead to signal absorption by the tissues or reflection back to the source [35]. There are many types of interactions that can possibly take place for different applications. In microwave imaging, reflection or scattering signals have been considered for cancer detection purposes [36]. As microwaves propagate through free space into a particular tissue, there is an interface layer between free space and the tissue medium. Different impedance of both mediums resulted in different microwave interaction for both tissues. These two mediums have different impedance values, and the microwaves will interact with both tissues differently. Some of the microwave signals will transmit through into the tissue and some will be reflected to the source or scattered away from it. The transmitted signal (*Et*) and reflected signal (*Er*) can be expressed as [37]:

𝐸𝑡 = 𝜏 ∙ 𝐸𝑖(9)

𝐸𝑟 = Γ ∙ 𝐸𝑖(10)

where *Ei* = incident wave, *τ* = transmission coefficient and Γ = reflection coefficient.

Two different mediums of reflection coefficient could be determined from their impedance values. Transmission line theory stated that wave reflection could occur when incoherence incident wave undergoes impedance characteristics changes. Thus, reflection coefficient is given as [38]:

(11) $$\Gamma =\frac{Z_{n}-Z_{m}}{Z_{n}+Z_{m}}$$

*μo* = permeability of free space, *εo* = permittivity of free space. For a medium ‘*m*’, permittivity *εm* is given as:

𝜀_𝑚_ = 𝜀_𝑟_, ∙ 𝜀_𝑜_ (12)

where *εm* = relative permittivity of the material.

Material impedance could be expressed as:

(13) $$Z_{m}=\sqrt{\frac{{µ}_{0}}{\varepsilon_{m}}}=\sqrt{\frac{{µ}_{0}}{\varepsilon_{r,m}\varepsilon_{0}}}=\frac{Z_{0}}{\sqrt{\varepsilon_{r,m}}}$$

Similarly, for medium ‘*n*’,

(14) $$Z_{n}=\frac{Z_{0}}{\sqrt{\varepsilon_{r,n}}}$$

Replacing Equations (13) and (14) for Equation (15), the reflection coefficient is given as:

(15) $$\Gamma ==\frac{\sqrt{\varepsilon_{r,m}}-\sqrt{\varepsilon_{r,n}}}{\sqrt{\varepsilon_{r,m}}+\sqrt{\varepsilon_{r,n}}}$$

On the other hand, transmission coefficient is given as:

(16) $$\tau =\frac{2\;\cdot \;\sqrt{\varepsilon_{r,m}}}{\sqrt{\varepsilon_{r,m}}+\sqrt{\varepsilon_{r,n}}}$$

**Figure 10:**
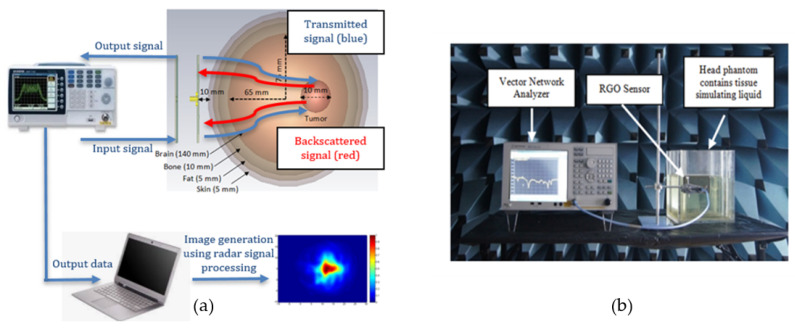
The diagram of the proposed brain tumor imaging system; (**a**) simulated, (**b**) measurement.

These Equations and analyses could be applied to all types of microwave imaging techniques including UWB radar imaging. As for the initial step in microwave imaging detection, UWB sensor reflections can determine the existence of abnormalities at tissue boundaries [37,38]. Transmitted and reflected signal ratio to the incident signal are known as scattering parameters. These parameters may be measured or calculated. They are measured in microwave imaging when a source sensor or an array sensor sequentially transmits microwave signals into a tissue of interest receive the reflected or transmitted signals [33]. Scattering parameters are better understood from the perspective of multiport networks or the array system of sensors. The simulated and experimental arrangement of the microwave imaging method to identify the brain tumor is illustrated Figure 10. The tumor is detected by the arrangements comprised of human head phantom, RGO sensor, and VNA.

**Figure 11:**
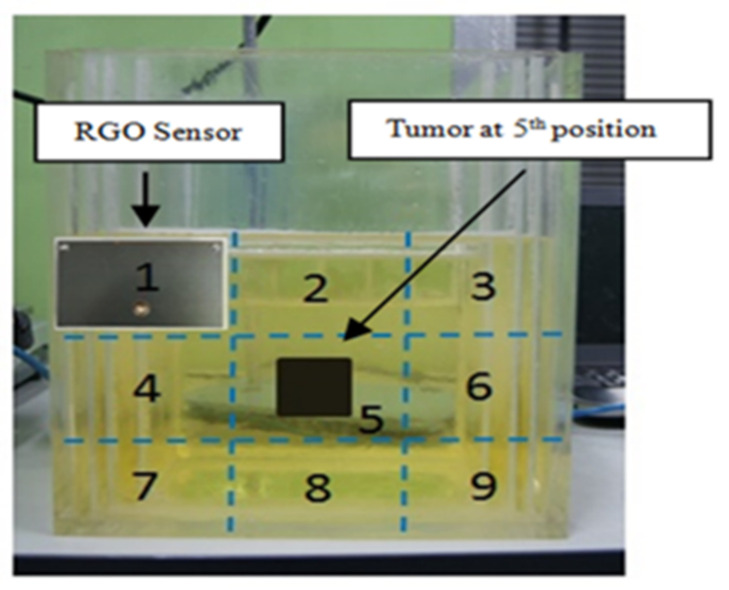
Human head phantom scanning area.

To achieve an accurate result, the measurements are performed in an anechoic chamber in order to remove the unwanted signal that might interfere with the result. The RGO sensor is placed parallelly 10 mm away from the head phantom for every reading in order to have optimum signal penetration [39]. The human head phantom used in this work consists of a four-layered rectangular structure sized at 194 × 164 × 260 mm^3^. Its shell is made of Plexiglas (*εr* = 2.3) and is partitioned into sections of brain, skull, fat, and skin. The brain tissue is placed in the innermost (center) of this phantom with an area of 170 × 140 mm^2^, followed by the skull (10 mm thick), fat (5 mm thick), and skin (5 mm thick). Each tissue is separated by a 1-mm-thick Plexiglas layer. A rectangular shape of the head phantom has been used due to the bending limitation of the Plexiglass structure. There are two different conditions in emitting the signal towards head phantom, namely with and without tumor, to acquire signal different values to be applied toward the confocal microwave radar-based algorithm to generate an image. Figure 11 shows the sensor-emitted microwave signal for nine specific regions in order to have full scanning of the entire one-sided region of the phantom where the tumor is located at region 5. The brain tumor is made of mixture of deionized water, polyethylene powder, aluminium powder and hydroxy-ethylcellulose to become a semi-solid structure to resemble the actual tumor, which has the permittivity value of 63.2 (similar to an actual brain tumor). The received backscattered signal (S11) from nine different regions of presence and absence of tumor are calculated, where the difference values acquired are then being converted using image processing for generating the image.

To acquire a clear image, averaging and interpolation steps have been added to the available original delay-and-sum algorithm for the enhancement. The purpose of averaging is to reduce the effects of messes originating from dominating reflections of environment, sensor, and equipment. At each frequency sample f and each sensor location *(v_k)* in (*f*, *k* ∈ *N*), the complex reflection coefficient matrix Γ (*j*, *k*) is calculated. A theoretical interpolation approach, on the other hand, is used to produce new data points in a series of known data points. In the meantime, interpolation helps to fill up the unfilled elements among the adjacent pixels by predicting based on the value of certain adjacent pixels [40].

Essentially, the energy at the brain’s focus point may be computed by [41]:

(17) $$E(x,\;y,\;z)=\int_{0}^{\tau }{\left(\sum_{1}^{M}y(t-T\;\left(x,\;y,\;z\right))\right)}^{2}\;\mathrm{dt},$$

where 𝑀 = total amount of received signal energy, 𝑦 = simulated received energy signal, 𝑇 = time-delay of each focal point, and 𝜏 = integration range. Assuming that the point of convergence is at (𝑥, 𝑦, 𝑧) and distance (𝑑) to sensor (𝑖), the time-delay can be communicated as [41]:

(18) $$d_{i}=\frac{\sqrt{{\left(x-x_{i}\right)}^{2}+{\left(y-y_{i}\right)}^{2}+{\left(z-z_{i}\right)}^{2}\;}}{v}$$

where *v* is the brain tissue spread velocity that could be additionally determined with [41]:

(19) $$v=\frac{\mu \varepsilon }{2}{\left\lfloor \sqrt{1+{\left(\frac{\sigma }{\omega \varepsilon }\right)}^{2\;}+1}\right\rfloor }^{0.5}$$

Since the distance between the port and the aperture centre at the sensor surface is limited, a time-delay must account for both values determined by the equation above and the time-delay taken by the port to aperture sensor surface. On the other hand, because of the tumor response constantly coming at low amplitude and always being associated with responses from other tissue, there is a need to extract the tumor response from other tissues’ response in order to have tumor response only for signal processing purposes. Priori method is used for the signal extracting purpose where signals are collected under two different conditions; absence of tumor within the human head phantom (reference signal) and when the tumor is placed within the head phantom. Both conditions of signals are subtracted from each other to have only the backscattered signal originating from the tumor located inside the human head phantom.

An averaging approach is used to eliminate artefacts originating from environmental reflections and sensor reverberation. Averaging is performed by taking an average of all the tumor-only backscattered responses and then subtracting this average from all the tumor responses, which could be stated mathematically by [42]:

(20) $$X[n]=b[n]\frac{1}{M}\sum_{i=1}^{M}b^{\prime }\left[n\right]$$

A sensor with number of positions (*M*) was used in collecting backscattered signals and b’[n] is the backscattered signal at each sensor position. On the other hand, the Hamming Window was applied in removing lobes at both side of the tumour response, and the Inverse Fast Fourier Transform was applied in converting the domain from frequency to time to obtain a temporal equivalent of the tumor response’s frequency-dependent scattering profiles. The introduction of a filter for smoothing technique is the modification proposed in IDAS imaging algorithm. The smoother lowess filter was applied for the purpose of improving visual information in a scatter plot with minimal additional cost on the plotting function [41]. Lowess, also known as “locally weighted scatter plot smooth”, applied a linear regression method using local weights to smooth the data. The smoothing process uses neighboring data points and a regression weighting function to determine smoothed value. This smoother was implemented in the IDAS using the MATLAB built-in mslowess function.

Synthetic focusing computes the intensity value of the focal points which are directly related to the pixel values in microwave images. This is achieved by first computing the time delays of the smoothed tumor response signals for time shifting purposes. The time delays are calculated for sensor distance toward each pixel points in image projection area and evaluated by [42]:

(21) $$D_{\left(x,y\right)}=2\sqrt{{\left(X-x_{i}\right)}^{2}+{\left(Y-y_{i}\right)}^{2}+h^{2}}$$

where *h* is the distance between sensor and tumor, *x_i_* and *y_j_* represents pixel points coordinate and *X* and *Y* define the sensor positions. Time delay at each pixel point is then:

(22) $$t_{\;(x,y)}=\frac{D_{\left(x,\;y\right)}}{c/\sqrt{\varepsilon_{r}}}$$

*D*(*x*,*y*) is the sensor distance to each pixel point, *c* is the speed of light, *εr* is the average relative permittivity of the medium, given as 40 for the propagation of UWB pulses in a head model [43].

The intensity value at each pixel point is derived through calculating smoothed da-ta values for each time, *t*(*x*,*y*). This is calculated for each pixel point as:

(23) $$I_{\left(x,y\right)}={\left[\sum_{y=1}^{r}\sum_{x=1}^{c}s\left[n\right]\times \left(t_{\left(x,\;y\right)}\right)\right]}^{4}$$

where *S*[*n*] is the tumor response on which smoothing has been performed, *r* is the total number of sensor positions in a row and *c* is the total number of sensor positions in a column.

## 4. Result and Discussion

**Figure 12:**
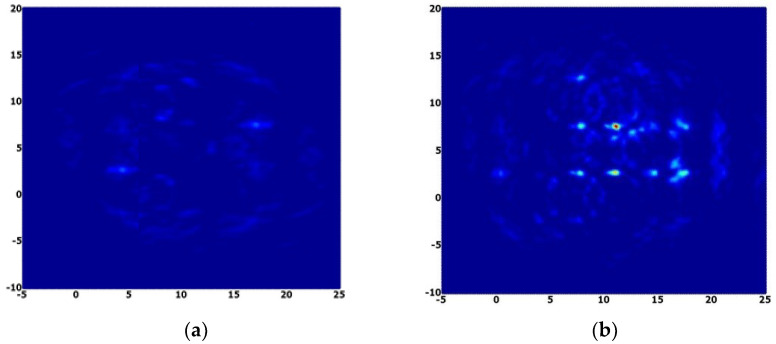
(**a**) Interpolated image and (**b**) averaged image.

**Figure 13:**
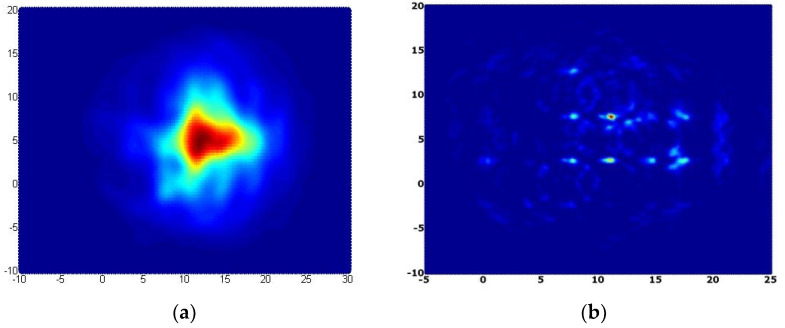
Final image; (**a**) with tumor, (**b**) without tumor.

Averaging and interpolation steps were applied in the original delay-and-sum algorithm to achieve better image clarity. In this manuscript, we are comparing the performance of the DAS algorithm technique between copper-based and Reduced Graphene Oxide (RGO) sensors for brain tumor detection where Refs. [13,40] used only the conventional copper sensor. Figure 12 shows the effects of interpolation and averaging algorithm during the process of producing the image. Averaging eliminates the messes effect originating from the direct reflections originated from the equipment and surrounding. Data points are made from the construction of interpolation of different data points from a discrete set. It assists the filling of the empty elements in between the adjacent pixels [34]. Figure 13 shows the images processed applied by the delay and sum algorithm in presence and absence of tumor.

Figure 13 clearly demonstrates that a microwave imaging system equipped with RGO sensor manages to produce a sharper and clearer image for both condition with tumor and without tumor as compared with another microwave imaging system equipped with a common sensor. This is due to the higher conductivity of graphene that leads to a higher signal produced by the RGO sensor to be radiated towards the tumor. The images demonstrated the efficiency of RGO sensor by perfect and clear images with better pixel intensity. The color with the most intensity (reddish-orange area) represents the presence of a tumor in the image because of the strongest scattering area that matches up to tumor presence in the human head phantom. Table 1 demonstrates the localization error of the detected tumor in the image as compared with the tumor’s actual position with the value of 0.2 cm or 2% of error which could be considered a tolerable error to prove the system is efficiently and accurately detecting the desired tumor.

## 5. Conclusions

This paper presents a reduced graphene oxide (RGO) array sensor to detect a tumor in a human brain phantom via a confocal radar-based microwave imaging technique. The RGO patches are fed using transmission lines and coupled to a parasitic element etched using conventional copper on a Taconic substrate. The sensor operated at frequencies of 1.2 GHz to 10.8 GHz within the ultra-wide band (UWB) frequency. The sensor demonstrates a high gain at 14.5 dB and has a size of 90 mm × 45 mm^2^. This system is operating experimentally by using an in-house-developed head phantom. The quality of the resulting image can be achieved successfully by improved delay and sum algorithm (IDAS). For future work, the presence of multiple tumors should be clearly investigated in a larger experimental region.

## Figures and Tables

**Table 1:** Tumor localization error.

	Actual Position		Position in Image		
	*X* (cm)	*Y* (cm)	*X*′ (cm)	*Y*′ (cm)	Localization Error (cm)
UWB sensor	10.0	5.0	10.2	5.2	0.2

## Data Availability

The data presented in this study are available on request from the corresponding author.
